# Adherence to vascular care guidelines for emergency revascularization of chronic limb-threatening ischemia

**DOI:** 10.1016/j.jvscit.2023.101299

**Published:** 2023-08-25

**Authors:** Toby P. Speirs, Eleanor Atkins, Mohammed M. Chowdhury, Diane R. Hildebrand, Jonathan R. Boyle

**Affiliations:** a Department of Vascular Surgery, Cambridge University Hospitals, Queens' College, Cambridge, UK; b Clinical Effectiveness Unit, Royal College of Surgeons of England, London, UK; c Department of Surgery, School of Clinical Medicine, University of Cambridge, Cambridge, UK; d School of Clinical Medicine, University of Cambridge, Cambridge, UK

**Keywords:** Amputation, Chronic limb-threatening ischemia, Diabetic foot, Intermittent claudication, Peripheral arterial disease

## Abstract

**Objective:**

In 2022, the National Health Service Commissioning for Quality and Innovation (CQUIN) indicator for vascular surgery, with its pay-for-performance incentive for timely (5-day) revascularization of chronic limb-threatening ischemia (CLTI), was introduced. We sought to assess its effects in terms of (1) changes in the care pathway process measures relating to timing and patient outcomes; and (2) adherence to the Peripheral Arterial Disease Quality Improvement Framework (PAD-QIF) guidelines for patients admitted with CLTI.

**Methods:**

A retrospective before-and-after cohort study was performed from January to June 2022 of nonelective admissions for CLTI who underwent revascularization (open, endovascular, or hybrid) at Cambridge University Hospitals National Health Service Foundation Trust, a regional vascular “hub.” The diagnostic and treatment pathway timing-related process measures recommended in the PAD-QIF were compared between two 3-month cohorts—before vs after introduction of the CQUIN.

**Results:**

For the two cohorts (before vs after CQUIN), 17 of 223 and 17 of 219 total admissions met the inclusion criteria, respectively. After introduction of financial incentives, the percentage of patients meeting the 5-day targets for revascularization increased from 41.2% to 58.8% (*P* = .049). Improvements were also realized in the attainment of PAD-QIF targets for a referral-to-admission time of ≤2 days (from 82.4% to 88.8%; *P* = .525) and admission-to-specialist-review time of ≤14 hours (from 58.8% to 76.5%; *P* = .139). An increase also occurred in the percentage of patients receiving imaging studies within 2 days of referral (from 58.8% to 70.6%; *P* = .324). The reasons for delay included operating list pressures and unsuitability for intervention (eg, active COVID-19 [coronavirus disease 2019] infection). No statistically significant changes to patient outcomes were observed between the two cohorts in terms of complications (pre-CQUIN, 23.5%; post-CQUIN, 41.2%; *P* = .086), length of stay (pre-QUIN, 12.0 ± 12.0 days; post-QUIN, 15.0 ± 21.0 days; *P* = .178), and in-hospital mortality (pre-QUIN, 0%; post-QUIN, 5.9%). Other PAD-QIF targets relating to delivery of care were poorly documented for both cohorts. These included documented staging of limb threat severity with the WIfI (wound, ischemia, foot infection) score (2.9% of patients; target >80%), documented shared decision-making (47.1%; target >80%), documented issuance of written information to patient (5.9%; target 100%), and geriatric assessment (6.3%; target >80%).

**Conclusions:**

The pay-for-performance incentive CQUIN indicators appear to have raised the profile for the need for early revascularization to treat CLTI, engaging senior hospital management, and reducing the time to revascularization in our cohort. Further data collection is required to detect any resultant changes in patient outcomes. Documentation of guideline targets for delivery of care was often poor and should be improved.

Chronic limb-threatening ischemia (CLTI) is “a clinical syndrome defined by the presence of peripheral artery disease (PAD) in combination with rest pain, gangrene[,] or lower limb ulceration >2 weeks [in] duration.”^1^ CLTI is increasing in prevalence and cost to healthcare services worldwide.^2^ It carries significant risk of mortality and limb loss.^3^ When clinically appropriate, early revascularization to alleviate ischemia reduces the risk of amputation and mortality.^3^^,^^4^

Lower limb amputation carries a high mortality rate, with a recent systematic review estimating 5-year mortality for any major lower limb amputation from 52% to 80%.^5^ Major amputation also carries a high 30-day readmission rate of 16.5%, according to the National Health Service (NHS) Hospital Episode Statistics data.^6^ During 2020 to 2021, nonelective amputations cost the NHS >£115 million.^7^ Avoiding these risks and resource costs is highly desirable.

In March 2018, the NHS “Getting It Right First Time (GIRFT) National Specialty Report for Vascular Surgery in the United Kingdom” was published.^6^ Unwarranted care variation was described, especially in the wait times for clinically urgent surgery. The report made several recommendations for service improvements, including an “increase in early availability of revascularization surgery” for lower limb ischemia to reduce amputation rates.

In March 2019 (updated March 2022), the Vascular Society for Great Britain and Ireland acted on the GIRFT report and published best practice guidelines for clinical care pathways of PAD in the form of the Peripheral Arterial Disease Quality Improvement Framework (PAD-QIF).^8^ The PAD-QIF lays out targets for the care of patients with CLTI—both emergently admitted and nonadmitted patients.

The PAD-QIF targets for nonelective admissions include the time from receipt of the referral to admission (≤2 days), from admission to cross-sectional imaging (≤12 hours), from admission to face-to-face review by a vascular surgeon (≤14 hours), and from admission to revascularization (whether open, endovascular, or hybrid; ≤5 days). These targets are widely seen to be challenging, requiring service reorganization to achieve them.^9^

In addition to the pathway time targets, further recommendations of the PAD-QIF include documenting the CLTI stage using the Society for Vascular Surgery WIfI (wound, ischemia, foot infection) score,^10^ shared decision-making, issuance of written information to the patient; and geriatric assessment pre- and postoperatively.

On April 1, 2022, the annual NHS Commissioning for Quality and Innovation (CQUIN) scheme was reintroduced after a 2-year hiatus during the COVID-19 (coronavirus virus disease 2019) pandemic.^11^ For 2022 to 2023, the scheme included an incentive for improvements in vascular surgery: nonelective CLTI patients should receive revascularization ≤5 days of admission. The CQUIN incentivizes improvements by offering pay for performance for adherence to specific clinical indicators, which are reviewed and published annually, thereby linking healthcare centers' income to quality improvement. Financial reimbursements to each arterial center are estimated at ≥£500,000, depending on NHS trust size. This incentivizes management in each center to support efforts to decrease the time to revascularization for increased funding.^12^ The expectation is that encouraging improvements in clinical processes will translate into improvements in patient outcomes. The CQUIN estimates nationwide annual savings of £12 million if delays in the pathway for emergency revascularization are reduced.^10^^,^^12^

Studies have generally drawn only limited conclusions about the long-term effects of pay-for-performance schemes such as the CQUIN. One study compared hospitals with (n = 207) and without (n = 406) pay-for-performance schemes in the United States and found that such schemes were associated with improvements in many quality measurements, especially in centers with low baseline performance.^13^ However, a recent systematic review found that pay-for-performance schemes improved care processes in ambulatory settings but showed little evidence of improving patient health in hospital settings.^14^

Generally, positive effects of pay-for-performance schemes are modest, short-lived, or inconsistent.^14^^,^^15^ Nonetheless, outcomes are often more positive in the United Kingdom than in the United States because of larger financial incentives.^14^

We sought to assess how well the targets specified in the PAD-QIF were being attained for patients undergoing emergency revascularization procedures for CLTI but also, specifically, whether a change had occurred in the attainment of the 5-day revascularization target after introduction of the CQUIN's financial incentive. This investigation was undertaken in a large vascular surgical hub—a hospital center with large resource and procedural capacity, receiving inpatients from the “spoke” hospitals throughout its region.

## Methods

### Setting

This retrospective before-after cohort study was conducted at the vascular surgery department at Addenbrooke's Hospital, Cambridge University Hospitals (CUH) NHS Foundation Trust. CUH provides secondary and tertiary vascular specialist care to a catchment area of ∼1.32 million patients in the east of England,^16^ acting as a hub for vascular service provision. The NHS Foundation Trust clinical audit department approved the present study.

### Study procedures

All patients admitted under the vascular surgery department from January 1, 2022 to June 30, 2022 were retrospectively assessed for inclusion (n = 442). The inclusion criteria were as follows:

• Nonelective (emergency) admission
• Diagnosis of CLTI (clinically coded or documented evidence of PAD with rest pain, ulceration, or gangrene with a duration >2 weeks, equivalent to a Rutherford score of 4, 5, or 6)
• Revascularization as intervention

Patients with acute limb ischemia were excluded. A total of 34 patients met the inclusion criteria. These patients were divided into two cohorts: those who underwent revascularization before the introduction of the 2022 to 2023 CQUIN (January 1 to March 31, 2022; n = 17) and those who underwent revascularization after the CQUIN introduction (April 1 to June 30, 2022; n = 17).

### Baseline data and outcomes

The electronic hospital records of these 34 patients were reviewed in full. The data extracted included the following:

• Baseline demographics (sex, age at admission, ethnicity; home postal code, comorbidities)
• Referral details (date, time, referral source)
• CLTI status (clinical code, symptoms, symptom duration)
• Admission details (date, time, reasons for delay)
• Imaging details (date of duplex ultrasound or cross-sectional imaging study)
• Specialist review (date, time, clinician grade, WIfI score, toe pressure or ankle brachial pressure index)
• Revascularization intervention (date, type [open, endovascular, hybrid], surgical outcome [success, failure, mixed], reasons for delay)
• Outcomes (discharge date, in-hospital mortality, complications)
• Decision-making and information governance (documented shared decision-making with the patient, interconsultant decision-making, documented provision of written information to the patient; review by a clinician in geriatrics department or department of medicine for the elderly [DME]).

The patient postal codes were used to assess the distance from the CUH. The cross-sectional imaging study was computed tomography angiography (CTA). If up-to-date imaging studies were performed at the referring center before admission to the CUH, the time from admission to the first imaging study was considered as 0 hours. For patients undergoing both CTA and duplex ultrasound on the same day, neither modality was considered as antecedent to the other. Specialist review was defined in concordance with the PAD-QIF as a “vascular surgeon ‘face to face’ review.”^8^ This definition includes consultants, registrars, and core surgical trainees in vascular surgery. Patients who underwent multiple procedures for CLTI during their admission had data extracted for their index procedure only and were included in the cohort during which the index procedure had occurred.

A “failure” outcome of revascularization was defined as no improvement in distal flow. A “mixed” outcome was defined as residual arterial stenosis. Complications were determined by the occurrence of major adverse cardiovascular or limb events (MACE/MALE) during the pertinent admission and were graded using the Clavien-Dindo classification. MACE were defined as stroke, myocardial infarction, death, hospitalization because of heart failure, and cardiac revascularization. MALE were defined as untreated loss of patency, reintervention on the index arterial segment, and amputation of the index limb.

An interconsultant discussion was defined as documentation of a formal or an informal discussion between vascular consultant clinicians. Shared decision-making with patients was considered as documentation of discussion with the patient about their wishes.

### Statistical analysis

Data were grouped as counts and percentages to one decimal place. The parametricity of the data was assessed using skewness, with Fisher's moment coefficient of skewness between −0.5 and 0.5 considered parametric. Averages were recorded as either the mean ± standard deviation for parametric data and the median and interquartile range for nonparametric data.

For categorical data, the χ^2^ test was used to compare the two cohorts. *P* values are presented to three decimal places. *P* ≤ .05 was considered statistically significant. To compare the mean values of the parametric variables, unpaired, two-tailed *t* tests were used. When assessing for correlation, the Spearman rank correlation coefficients were calculated. Statistical analysis was performed using the Analysis ToolPak for Microsoft Excel, version 2211 (Microsoft Corp).

## Results

### Demographics

The pre-CQUIN and post-CQUIN cohorts were both predominantly male (82.4% and 64.7%, respectively) and White (76.5% and 82.4%, respectively; Table I). The mean age was 72.9 ± 6.7 years and 68.8 ± 14.6 years for the pre- and post-CQUIN cohorts, respectively.

**Table I:** Patient demographics and comorbidities

Variable	All patients (n = 34)	Pre-CQUIN cohort (n = 17)	Post-CQUIN cohort (n = 17)
Male sex	25 (73.5)	14 (82.4)	11 (64.7)
White ethnicity^a^	27 (79.4)	13 (76.5)	14 (82.4)
Age, years	70.8 ± 11.7	72.9 ± 6.7	68.8 ± 14.6
Diabetes mellitus (type 1 or 2)	16 (47.1)	7 (41.2)	9 (52.9)
Hypertension	20 (58.8)	10 (58.8)	10 (58.8)
Current or ex-smoker	18 (52.9)	11 (64.7)	7 (47.1)
Ischemic heart disease	13 (38.2)	6 (35.3)	7 (41.2)

*CQUIN,* Commissioning for Quality and Innovation.

Data presented as number (%) or mean ± standard deviation.

a Non-White patients were of unknown ethnicity from the medical notes.

Across all patients, 73.5% were men, 79.4% were White (others of unknown ethnicity), and the mean age was 70.8 ± 11.7 years. The mean number of documented comorbidities or risk factors per patient was 7.8 and 7.3 for the pre-CQUIN and post-CQUIN groups, respectively. The frequency of the most common comorbidities is compared in Table I for the two cohorts. No statistically significant difference was found in the prevalence of any comorbidity between the two cohorts.

### Referral

Most admissions to the CUH were by transfer from the outlying centers throughout the East of England region (61.8%). One half of all these referrals were from secondary or tertiary services; however, many also came from accident and emergency presentations (35.3%). One patient was referred from a general practice appointment in primary care. The remainder were of unknown referral origin (n = 4).

### Admission

The median time from referral to admission was 0.0 ± 1.3 days (pre-CQUIN, 0.0 ± 2.0 days; post-CQUIN, 0.0 ± 1.0 day). After introduction of the CQUIN, the percentage of patients admitted within 2 days of referral increased mildly from 82.4% to 88.2% (*P* = .525; Fig). No relationship was found between the distance from the home address to the hub hospital and the time to admission from referral (Spearman correlation coefficient, 0.11).

**Fig:**
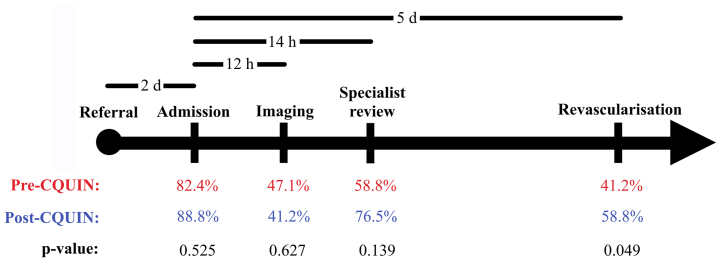
Percentage attainment rate of Peripheral Arterial Disease Quality Improvement Framework (PAD-QIF) time targets for admitted, nonelective patients. *Upper black bars* indicate PAD-QIF time targets for different points of the care pathway (*black arrow*). Percentage attainment of pre-Commissioning for Quality and Innovation (CQUIN) cohort (n = 17) compared with post-CQUIN cohort (n = 17) displayed, with *P* values of differences between percentage attainment rates.

### Imaging studies

All except for two patients received imaging (duplex or CTA, or both) before intervention (94.1%), with the two exceptional patients proceeding directly to intervention. Duplex ultrasound imaging was performed for 85.3% of all patients, which remained stable after the introduction of CQUIN (pre-CQUIN, 88.2%; post-CQUIN, 82.4%; *P* = .452). CTA was performed for 73.5% of all patients, which also remained stable (pre-CQUIN, 64.7%; post-CQUIN, 82.4%; *P* = .128).

The proportion of patients receiving CTA as the first imaging choice increased, although the difference was not statistically significantly (pre-CQUIN, 41.2%; post-CQUIN, 52.9%; *P* = .324). Two patients received both duplex ultrasound and CTA on the same day, and two patients proceeded to intervention without imaging studies.

Imaging studies were performed ≤12 hours of admission for fewer than one half of all patients (44.1%). However, most patients received imaging ≤2 days of referral (64.7%) with this increasing after the CQUIN from 58.8% to 70.6% (*P* = .324).

### Specialist review

Most patients were seen by a vascular surgery doctor ≤14 hours of admission (71.9%). Again, more patients met this target after introduction of the CQUIN, increasing from 58.8% to 76.5% (*P* = .139; Fig). One half of the patients were reviewed at the first encounter by a vascular consultant; the remainder were reviewed by a vascular registrar, with the exception of one patient, who was first reviewed by a core surgical trainee. The WIfI score was documented for only a single patient before the CQUIN (2.9%). Most patients had either toe pressures or ankle brachial pressure index documented (70.6%).

### Revascularization

The proportion of patients meeting the 5-day target for admission to revascularization increased after the introduction of the CQUIN, from 41.2% to 58.8% (*P* = .049; Fig). The mean time from admission to revascularization for pre-CQUIN and post-CQUIN was 6.8 ± 3.9 days and 4.6 ± 2.2 days, respectively. One procedure (2.9%), after the CQUIN, was performed outside of normal operating hours (Monday-Friday, 9:00 am to 5:00 pm).

The intervention types included open (pre-CQUIN, 47.1%; post-CQUIN, 23.5%; *P* = .05), endovascular (pre-CQUIN, 41.2%; post-CQUIN, 41.2%; *P* = 1.00), and hybrid (pre-CQUIN, 11.8%; post-CQUIN, 35.3%; *P* = .002). Most revascularization interventions resulted in success or mixed success (94.1%), with the rates identical between the pre-CQUIN and post-CQUIN cohorts.

### Outcomes

The median length of stay was 13.5 ± 17.0 days. After the introduction of the CQUIN, the median length of stay increased from 12.0 ± 12.0 days to 15.0 ± 21.0 days (*P* = .178). The incidence of complications (MACE/MALE) was also higher after the CQUIN (pre-CQUIN, 23.5%; post-CQUIN, 41.2%; *P* = .086). Approximately one third of all patients (32.4%) experienced postoperative complications. However, only 11.8% of all patients required surgical, endoscopic, or radiologic intervention (Clavien-Dindo grade of ≥IIIa). One post-CQUIN patient (5.9%) underwent a major limb amputation during their admission. In-hospital mortality was 0% before the CQUIN and 5.9% after (n = 1).

### Decision-making and information governance

Documented evidence of shared decision-making with patients was found for 47.1% of cases and interconsultant discussion for 41.2%. Documented provision of written information was present for 5.9% of patients (n = 2), both before the CQUIN. A review pre- or postoperatively by a clinician in the geriatrics department or DME was also evident for 6.3% of eligible patients (2 of 32 patients), both after the CQUIN. The attainment rates of PAD-QIF guideline targets are shown in the Fig and Tables II and III. The time-related process measures were compared between the pre- and post-CQUIN cohorts because the CQUIN indicators are related to pathway timelines.

**Table II:** Attainment of timeline-related Peripheral Arterial Disease Quality Improvement Framework (PAD-QIF) guidelines for care of admitted patients with chronic limb-threatening ischemia (CLTI) for revascularization

PAD-QIF guideline target	All patients (n = 34)	Pre-CQUIN cohort (n = 17)	Post-CQUIN (n = 17)	*P* value
Referral to admission of ≤2 days	29 (85.3)	14 (82.4)	15 (88.8)	.525
Admission to imaging study of ≤12 hours	15 (44.1)	8 (47.1)	7 (41.2)	.627
Admission to specialist review ≤14 hours	23 (67.6)	10 (58.8)	13 (76.5)	.139
Admission to revascularization of ≤5 days^a^	17 (50.0)	7 (41.2)	10 (58.8)	.049

*CQUIN,* Commissioning for Quality and Innovation.

Data presented as number (%).

a Pre-CQUIN and post-CQUIN cohorts compared because the CQUIN sought to incentivise time-related targets.

**Table III:** Attainment of nontimeline Peripheral Arterial Disease Quality Improvement Framework (PAD-QIF) guidelines for patients with chronic limb-threatening ischemia (CLTI) admitted for revascularization

PAD-QIF guideline	Target, %	All patients (n = 34)	Pre-CQUIN cohort (n = 17)	Post-CQUIN cohort (n = 17)
Severity of limb threat staged using SVS WIfI score	>80	1 (2.9)	1 (5.9)	0 (0.0)
Shared decision-making with evidence in medical record >80%	>80	16 (47.1)	10 (58.8)	6 (35.3)
Written information given to patients	100	2 (5.9)	2 (11.8)	0 (0.0)
Specialists trained in CGA available to review eligible patients (n = 32) pre- and postoperatively	>80	2 (6.3)	0 (0.0)	1 (11.8)

*CGA,* Comprehensive geriatric assessment; *CQUIN,* Commissioning for Quality and Innovation; *SVS,* Society for Vascular Surgery; *WIfI,* wound, ischemia, foot infection.

Data presented as number (%).

## Discussion

### Demographics

The baseline demographics (sex, ethnicity, age) were comparable between the pre-CQUIN and post-CQUIN cohorts. The patients had a high burden of comorbidity, which likely contributed to the high complication rate, with 32.4% experiencing MACE/MALE. This was comparable to the complication rate seen in the wider literature. A recent large study of 7651 revascularized CLTI patients showed an in-hospital MACE/MALE rate of 32.1% using the same definitions as in the present study.^3^

The social determinants of health, including educational and socioeconomic status, in conjunction with demographics, have a probable effect on outcomes. Black race and low socioeconomic status have been associated with an increased prevalence and poorer outcomes of PAD.^17^, ^18^, ^19^ These factors could affect the lateness and severity of symptomatic presentation and, therefore, surgical outcomes. Access to data regarding social factors was limited in this patient cohort, and such an analysis was not our objective. The effect of social determinants on CLTI interventional outcomes represents a topic for further research.

### Care pathway

The CUH is part of a hub-and-spoke vascular network covering a large geographic area. It admitted most of its CLTI patients from other centers throughout the region. The CUH runs a daily “hot clinic” for patients with suspected CLTI who are referred from general practice and are suitable for management on an outpatient basis. This limits the number of patients admitted as an emergency after referral from the community. There was only one such patient in our cohort. Patients whose local hospital is a spoke are less likely to be able to be managed on an outpatient pathway because of transport needs, which might account for the greater proportion of patients from spoke hospitals being admitted for nonelective treatment. Previous work has also found that most CLTI admissions are from spoke centers.^4^

The patients in our cohort who presented to the accident and emergency department (35.3%) might have delayed seeking attention in primary care or their symptoms might have passed unrecognized before reaching a crisis point. It follows that these patients would have more advanced symptoms and would be more likely to be admitted for emergency management.

Regarding patient progression through the care pathway, most patients are admitted rapidly after referral (85.3% within the PAD-QIF target of 2 days), including patients transferred from spoke hospitals. Most patients were also then reviewed in a timely manner (71.9% within the PAD-QIF target of 14 hours). After the CQUIN, more patients were reviewed rapidly by a vascular surgery doctor (58.8% vs 76.5% within 14 hours; *P* = .139).

The 2018 GIRFT report suggested that admissions after referrals from other departments, even internally, could be a cause of delays^6^; however, this did not appear to be the case in our study, in contrast to national data.^4^ When the reasons for any delay to admission were documented, they included the need for medical optimization or imaging studies before transfer and COVID-19 infection.

The PAD-QIF target met least frequently in the CUH pathway was the time to imaging (44.1% within 12 hours). Imaging within 12 hours of admission is a challenging target for any center to attain.^9^ Achieving early imaging represents a point for improvement because suitable radiologic investigations inform the decisions about the intervention, facilitating timely treatment. A notable decrease occurred in the use of duplex ultrasound as the first-line imaging study, with an increase in CTA, after the CQUIN was introduced. This might indicate greater availability of CTA as an imaging modality, especially outside normal working hours.

The financial incentive of the CQUIN did appear to encourage rapid revascularization, given the statistically significant improvement in the 5-day target attainment (41.2% vs 58.8%; *P* = .049). This was the only pathway time target that changed to a statistically significant extent after the introduction of the 2022 to 2023 CQUIN, and it could be argued that the time to revascularization is the most important time metric for improving patient mortality and limb salvage outcomes.^4^ The tangible reasons for delay to revascularization included COVID-19 infection, medical optimization for surgery, and the lack of capacity on emergency operating lists.

No change occurred in the formal local protocols with the introduction of the CQUIN. Rather, it is posited that the improvement resulted from a change in attitude and culture by vascular clinicians and hospital management, with greater awareness of the need to perform revascularization rapidly, supported by national publicity surrounding the initiative. The CQUIN raised the profile of this need by engaging senior management through a financial incentive to support the vascular surgeons. Support of hospital management teams has proved vital in other forms of quality improvement in UK surgical contexts.^20^^,^^21^ Financial incentives have been successful in improving quality in other contexts, and this might be the mechanism by which the CQUIN has been effective.^13^

### Outcomes

The in-hospital mortality rate was 2.9% overall—a single patient in the post-CQUIN cohort died during admission. This is comparable to the in-hospital mortality rate reported previously in the literature for revascularized CLTI patients.^3^^,^^22^

Although attainment of the 5-day revascularization target increased, this did not translate into observable improvement in the short-term patient outcomes measured in our cohort. The length of stay, complication rates, and in-hospital mortality were all higher for the post-CQUIN cohort, although none of the differences reached statistical significance. This might reflect disease complexity, potentially signposted by an increase in the prevalence of more complex hybrid procedures performed in this cohort or that less time is available for preoperative optimization after CQUIN. Only one patient received revascularization outside of normal operating hours, which could not account for the differences in outcomes. The short-term outcome data available for this small cohort are a limitation of this study, and more long-term data from larger cohorts are required to evaluate the effects of the CQUIN over time.

### Other PAD-QIF guidance

Although the adherence to the PAD-QIF target timing-related process measures was generally good, the documentation of other targets related to the delivery of care was often poor. The WIfI scores, issuance of patient written information, and review by the geriatrics department or DME were infrequently reported. None of these met the goals for the attainment rates set out in the PAD-QIF (Table III).

For unknown reasons, a decrease occurred in the documentation of shared decision-making after the CQUIN. The CQUIN provides incentives only for timely revascularization of CLTI. Thus, although it is hoped that this might raise awareness of the PAD-QIF as a whole, including proper documentation, this is not a direct sequela of the financial incentive. One might reasonably expect an improvement in the documentation of shared decision-making, not a worsening. The dearth of documentation suggests that, although the CQUIN might improve adherence to time targets for treatment, it has not raised awareness of other PAD-QIF targets.

A large proportion (70.6%) of the cohort were aged >65 years and, thus, would have been suitable for review by a clinician in the geriatrics department or DME. However, this only occurred for 6.3% (n = 2) of the eligible patients. The value of a geriatrician review is highlighted in the literature by the decreased interventional success and survival rates among older patients with CLTI.^23^ An attentive postoperative medication review and adherence improves long-term survival.^23^ The geriatric nutritional risk index and patient age and activity are strong predictors of amputation-free survival,^24^, ^25^, ^26^ and a perioperative comprehensive geriatric assessment can assist in mitigating these risks. A comprehensive geriatric assessment might increase referral to other services and/or physicians and improve discharge planning,^27^ which might even reduce the length of stay.

Many of these processes, such as WIfI scoring and shared decision-making are likely performed regularly in the clinical setting but also are not being documented well. Recording these scores, events, and conversations in the patient notes is a point for improvement. The use of the WIfI score is endorsed by the joint Global Vascular Guidelines for the management of CLTI.^1^ Uniform scoring systems enable objective quantification of disease to track its progression, allow for clearer transfer of care, and permit effective epidemiologic research. Shared decision-making in the management of PAD is recommended, not only by the Vascular Society for Great Britain and Ireland PAD-QIF guidance, but also by numerous other international guidelines.^1^^,^^28^, ^29^, ^30^ Patients generally desire to be well-informed about their treatments, and their involvement in decision-making increases their satisfaction.^31^^,^^32^

Local protocols and forms should be updated to encourage documentation. Where such structures exist, documentation is strengthened. The measurement of toe pressures is a standardized procedure performed by vascular scientists with written reports. These were documented well in the notes. In contrast, the forms for admitting new CLTI patients during this period at the CUH did not include the WIfI scores, discouraging their documentation.

### Study strengths and limitations

This is a single-center study of the early effects of a national pay-for-performance program to reduce the time to revascularization for patients admitted with CLTI. To the best of our knowledge, no reported evidence is currently available on this subject. The cohorts were naturally of a small sample size; however, despite this, statistical significance was reached for the primary outcome measure of the time to revascularization across comparable cohorts.

The small sample sizes and relatively short study period likely contribute to the lack of statistically significant changes in patient outcomes and individual timing-related process measures. The before-and-after design of the study lacks a control cohort that could account for secular trends;; thus, our results could be skewed by fluctuations in pressure on the CUH vascular service. However, in the context of the national CQUIN program, a control cohort would be difficult to obtain.

The lack of documented scores such as the WIfI score on admission restricted our ability to assess the severity of presentation in a standardized manner. Therefore, differences in initial clinical severity between cohorts could have influenced patient outcomes.

Another limitation of the present study is the short follow-up period. Long-term patient outcomes and any relationship of this with revascularization delays were not analyzed beyond the relevant admission for revascularization. Long-term outcomes after revascularization and the effect of the introduction of the CQUIN on these is a question for future research.

## Conclusions

The results of our single-center before-and-after retrospective cohort study suggest that the financial incentive of the vascular CQUIN in 2022 to 2023 has been effective in engaging hospital management, allowing vascular surgeons to reduce the time to revascularization for emergent CLTI. The 5-day target for CLTI revascularization is becoming embedded in practice in England. This has improved other timing-related pathway points. Further data with longer follow-up are required to detect any resultant improvements in patient outcomes. Our data suggest that the introduction of the CQUIN, although based on the PAD-QIF, has not improved the adherence to PAD-QIF targets related to WIfI scoring, geriatric assessments, issuance of written information to patients and shared decision-making. These targets are currently documented poorly, representing an area for clinical improvement.
